# Predictors of Heart Rate Depression During Carotid Artery Stenting in Presumed Low-Risk Patients: A Retrospective Single-Center Observational Study

**DOI:** 10.3390/jcm15124832

**Published:** 2026-06-22

**Authors:** Itamar Gothelf, Farouq Alguayn, Galia Karp, Krestina Shihada, Yair Zlotnik, Yana Mechnik Steen, Anat Horev

**Affiliations:** 1 Faculty of Health Sciences, Ben-Gurion University of the Negev, Beer-Sheva 8410501, Israel; 2 Department of Neurosurgery, Soroka University Medical Center, Beer-Sheva P.O. Box 151, Israel; 3 Department of Anesthesia, Soroka University Medical Center, Beer-Sheva P.O. Box 151, Israel; 4 Department of Neurology, Soroka University Medical Center, Beer-Sheva P.O. Box 151, Israel

**Keywords:** carotid artery stenting, hemodynamic depression, bradycardia, hypotension, risk factors

## Abstract

**Background:** Hemodynamic depression, characterized by bradycardia and hypotension, is a common complication of carotid artery stenting (CAS) and is primarily attributed to carotid sinus baroreceptor stimulation. While prophylactic atropine is often used in high-risk patients, predictors of unexpected hemodynamic depression among patients initially deemed low-risk remain incompletely defined. **Objective:** To identify clinical, anatomical, and procedural predictors of hemodynamic depression in patients undergoing CAS without prophylactic atropine. **Methods:** We performed a retrospective, single-center observational study of consecutive patients undergoing CAS between January 2015 and May 2024. Patients who received prophylactic atropine for low baseline heart rate (HR) were excluded. Hemodynamic depression was defined as a >20% reduction in HR from baseline. Absolute bradycardia (HR <50 bpm) and hypotension (>40% reduction in systolic blood pressure) were recorded descriptively. **Results:** A total of 158 patients underwent CAS, of whom 33 (20.9%) were excluded due to prophylactic atropine administration for low pre-procedural heart rates (<60 bpm). Among 125 included patients, 62 (49.6%) experienced significant HR reduction during CAS. In multivariable analysis, a shorter distance between the stenotic lesion and the carotid bifurcation was independently associated with hemodynamic depression (OR 0.90 per mm increase; 95% CI 0.82–0.99; *p* = 0.023). Greater intraprocedural reductions in systolic and mean arterial pressure were also associated with HR depression. Traditional clinical risk factors, including age, sex, comorbidities, degree of stenosis, calcification severity, anesthesia type, and procedure urgency, were not independently predictive. **Conclusions:** Hemodynamic depression remains frequent during CAS even among patients classified as low risk. Lesion proximity to the carotid bifurcation is a key anatomical predictor of autonomic instability, highlighting the limitations of standard risk stratification and supporting a lesion-specific approach to periprocedural hemodynamic management.

## 1. Introduction

Carotid artery stenosis remains a major cause of ischemic stroke, accounting for a significant proportion of cerebrovascular morbidity and mortality worldwide. While carotid endarterectomy (CEA) has long been the gold standard for severe carotid stenosis, carotid artery stenting (CAS) has emerged over the past two decades as a less invasive alternative [1,2]. CAS is performed across a range of clinical settings: emergently as an adjunct to mechanical thrombectomy during acute ischemic stroke, semi-emergently for symptomatic high-grade stenosis, or electively for critically stenotic lesions identified during routine evaluation [3,4].

A frequent complication of CAS is hemodynamic instability (HI), typically presenting as hypotension and/or bradycardia, most often during or immediately after balloon angioplasty and stent deployment [5]. Reported incidence rates vary widely, from 7% to 80%, likely reflecting procedural differences, patient selection, and variation in definition across studies [6,7]. The primary mechanism appears to be mechanical stimulation of the carotid sinus baroreceptors, triggering exaggerated parasympathetic responses [8]. In severe cases, this may cause profound bradycardia, transient asystole, or significant hypotension, necessitating urgent pharmacologic support [9]. Other major complications of CAS include stroke, transient ischemic attack, and myocardial infarction [10].

Prophylactic administration of atropine or vasopressors before balloon inflation and stent deployment has been proposed to attenuate reflex-mediated hemodynamic depression [11]. However, indiscriminate use of these agents may provoke peri-operative cerebral hyperperfusion, posing a serious risk of intracerebral hemorrhage or neurologic deterioration, and may also expose patients to avoidable cardiovascular complications, including arrhythmias or myocardial ischemia [12,13]. Thus, accurately identifying patients at high risk for hemodynamic instability is critical to optimize prophylactic strategies while minimizing unnecessary interventions.

Although prior studies have suggested associations between patient characteristics—such as age, sex, smoking history, diabetes, recent stroke, and asymptomatic stenosis—and the occurrence of HI, results have been inconsistent, and some studies reflect outdated clinical data [8,14,15,16,17]. Prior studies have reported that lesion-specific and physiologic factors are associated with increased susceptibility to periprocedural hemodynamic depression [18,19]. Notably, a recent large-scale meta-analysis identified a wide array of predictors, including diabetes, stenosis-to-bifurcation <10 mm, carotid bulb involvement, calcified or eccentric plaque, severe or contralateral stenosis, and stent design [18]. However, these broad datasets reflect highly heterogeneous populations. Specifically, they include patients with baseline bradycardia or those receiving routine prophylactic interventions. To date, data focusing exclusively on patients clinically classified as ‘low-risk’ remain scarce. This study addresses this gap by isolating a strictly curated cohort of patients without baseline bradycardia who did not receive prophylactic atropine, aiming to identify predictors of unexpected, pure vagal response.

Recent advances in procedural techniques, stent design, and embolic protection devices have improved the safety profile of CAS; however, hemodynamic complications remain a persistent challenge. Understanding patient-specific and procedural predictors of HI is crucial not only for procedural planning but also for developing tailored prophylactic strategies that can enhance patient outcomes and reduce perioperative morbidity.

This study focuses specifically on patients initially deemed at low risk for hemodynamic instability who did not receive prophylactic atropine. By analyzing the untreated, presumed low-risk group, we aimed to identify predictors of unexpected hemodynamic depression, providing practical insights to refine prophylactic strategies and improve real-time decision-making during carotid artery stenting.

## 2. Materials and Methods

### 2.1. Study Population

This retrospective, observational study was conducted at a single tertiary medical center and included 158 consecutive patients who underwent carotid artery stenting (CAS) between January 2015 and May 2024. Eligible participants were categorized as follows: (1) Asymptomatic, elective patients with stenotic lesions > 80% who were considered at low procedural risk (defined as a complication risk < 3%) [20,21]; these patients were admitted electively and treated with DAPT for 7 days prior to the procedure. (2) Symptomatic, semi-emergent patients who presented with ipsilateral hemispheric symptoms or a transient ischemic attack and had proximal ICA stenosis > 70%; these patients were admitted following symptom onset and treated with DAPT for a minimum of 5 days prior to the procedure [22]. (3) Patients presented within 24 h of symptom onset with disabling anterior-circulation ischemic stroke (NIHSS > 6) requiring endovascular therapy. Angiography demonstrated a severe ipsilateral cervical ICA stenosis or occlusion with a concomitant intracranial large-vessel occlusion, consistent with a tandem lesion. In most cases, intracranial mechanical thrombectomy was performed during the same session; however, if no intracranial occlusion was identified following CAS, thrombectomy was not pursued. The decision to proceed with emergent carotid artery stenting —either prior to thrombectomy to facilitate intracranial access or following intracranial reperfusion—was made intra-procedurally based on lesion severity, feasibility of device passage, and real-time flow assessment [22]. In all cases, the decision to perform CAS was made by a multidisciplinary team consisting of a radiologist, stroke neurologist, and interventional neuroradiologist.

The study included consecutive patients who underwent CAS during the specified period, with the single exception of individuals who received prophylactic atropine prior to stenting due to a baseline heart rate of <60 beats per minute. By excluding patients with pre-existing baseline bradycardia, the remaining cohort represents a relatively low-risk population regarding predictable intra-procedural hemodynamic collapse. This threshold is conventionally used to define bradycardia and is commonly applied in clinical practice due to concern for intraprocedural heart-rate decline related to carotid sinus stimulation. Furthermore, all included patients underwent a routine pre-procedural electrocardiogram to rule out acute ischemic changes or high-grade conduction abnormalities, and laboratory screenings confirmed that baseline serum electrolyte levels, including potassium, calcium, and magnesium, were within normal limits prior to the intervention. All other patients who underwent CAS during the study period were included.

### 2.2. Procedural Details

For patients treated under Group 1 or Group 2 indications, carotid artery stenting (CAS) was performed awake under local anaesthesia. Following femoral arterial access, patients received unfractionated heparin (50 units/kg) targeting an ACT of 250–300 s. An 8F Neuron MAX guiding catheter (Penumbra, Alameda, CA, USA) was advanced to the common carotid artery, and an embolic protection device (SPIDER 5, Medtronic, Minneapolis, MN, USA) was deployed in the petrous ICA using a 0.014-inch microwire (Synchro, Stryker, Kalamazoo, MI, USA).

Stenting was performed at the operator’s discretion using CGuard (InspireMD, Tel Aviv, Israel), Xact (Abbott Vascular, Santa Clara, CA, USA), or Precise stents (Cordis, Miami Lakes, FL, USA), followed by post-dilatation with a Viatrac 5 × 20 mm balloon (Abbott Vascular, Santa Clara, CA, USA). Immediately prior to deployment, patients received 150 mg IV aspirin, followed by intra-arterial eptifibatide (2–3 mg) per institutional protocol.

Post-procedurally, patients were monitored in the neurointensive care unit overnight, followed by ward observation for a minimum of two additional days. All patients were discharged on dual antiplatelet therapy (aspirin and clopidogrel) for six weeks. At seven weeks, Doppler ultrasound assessed stent patency; clopidogrel was discontinued if flow was normal, and aspirin was continued.

For patients treated under Group 3 (emergent) indications, procedures were performed under general anesthesia. CAS was undertaken when angiography demonstrated a severe ipsilateral cervical ICA stenosis or occlusion with a concomitant intracranial large-vessel occlusion (tandem lesion). Once the decision to proceed was made, patients received heparin (50 units/kg) to achieve ACT > 250 s and IV aspirin (300–500 mg, weight-adjusted). CAS was performed before or after mechanical thrombectomy, depending on lesion severity, access feasibility, and real-time flow assessment.

Following the procedure, a non-contrast head CT was obtained. If intracranial hemorrhage was excluded, patients received intra-arterial eptifibatide (2–3 mg, weight-adjusted) and ticagrelor 90 mg via nasogastric tube, according to the center’s emergent stenting protocol [23].

An anesthesiologist was present in all procedures. In response to intraprocedural bradycardia during stenting or balloon angioplasty, and if HR did not recover spontaneously within 5 s, standard treatment included 0.5 mg Atropine, usually resulting in rapid normalization [19].

### 2.3. Data Collection

Clinical data were retrospectively extracted from electronic medical records and included baseline demographics, radiologic and procedural characteristics, as well as clinical outcomes. Hemodynamic depression was defined as a reduction in HR greater than 20% from baseline. Intra-procedural hypotension was defined as a decrease in systolic blood pressure (SBP) of more than 40%, and intra-procedural bradycardia was defined as HR less than 50 beats per minute. These criteria were established in accordance with thresholds commonly reported in the literature [15,24]. Radiologic assessments included the Alberta Stroke Program Early CT Score (ASPECTS), used to quantify the extent of early ischemic changes on baseline brain imaging in patients presenting with acute ischemic stroke, and the Woodcock calcification score, which was used to grade the severity of carotid artery calcification based on angiographic imaging. Additional imaging variables included the distance of the stenotic lesion from the carotid bifurcation, cervical level of stenosis, percentage of carotid stenosis (according to NASCET criteria), and lesion length. All radiologic measurements were assessed by a board-certified radiologist.

Changes in systolic blood pressure and mean arterial pressure (ΔSBP and ΔMAP) were calculated as the difference between baseline values and the minimum measurements recorded during the procedure.

### 2.4. Statistical Analysis

Descriptive statistics were utilized to summarize patient demographics, imaging and procedural features, clinical outcomes, perioperative complications, and hemodynamic measurements. Continuous data were expressed as mean ± standard deviation when normally distributed, or as median with interquartile range (IQR) when distribution was non-normal. Categorical data were reported as counts and percentages.

To examine associations between demographic, clinical, and radiologic variables and hemodynamic depression, univariate analyses were performed using the chi-square test or Fisher’s exact test for categorical variables, and the independent-samples *t* test or Mann–Whitney U test for continuous or ordinal variables, as appropriate based on data distribution. To identify independent predictors of peri-procedural hemodynamic depression among patients undergoing carotid artery stenting, a multivariable binary logistic regression analysis was subsequently executed using the standard enter method. Predictors were selected for inclusion based on clinical relevance and a trending significance in the univariate screening. The goodness-of-fit and calibration of the final regression model were characterized using the Omnibus Tests of Model Coefficients and Nagelkerke’s R^2^, while the model’s discriminative ability was reported via the overall classification accuracy. All statistical analyses were performed using SPSS software (version 29; IBM Corp., Armonk, NY, USA), with statistical significance defined as a two-sided *p*-value ≤ 0.05.

## 3. Results

A total of 158 patients underwent CAS, of whom 33 (20.9%) were excluded from the analysis after receiving prophylactic atropine at the operator’s and anesthesiologist’s discretion for persistently low pre-procedural heart rates (<60 bpm). Baseline characteristics of the study population, including comorbidities and chronic medications, are presented in Table 1 Univariate analysis comparing patients with and without significant post-procedural heart rate depression (<20% vs. >20% HR decrease) revealed that a history of hypertension (*p* = 0.015), chronic use of ACE-inhibitors (*p* = 0.024), and premorbid functional status (mRS) (*p* = 0.023) differed significantly between the two groups.

Anatomical, radiological, and procedural characteristics are summarized in Table 2. The indication for most procedures was symptomatic proximal internal carotid stenosis over 70%, with the majority classified as semi-emergent, followed by elective and emergent cases. Local anesthesia was the predominant anesthetic technique, and the Exact stent was the most frequently used device. Univariate analysis comparing patients with and without significant post-procedural heart rate depression (<20% vs. >20% HR decrease) revealed that post-procedural hypotension (*p* = 0.001) and a shorter distance from the stenosis to the carotid bifurcation (*p* = 0.018) were significantly associated with a >20% heart rate decrease.

Univariate analysis (Table 3) identified several predictors of a significant reduction in HR, defined as a >20% decrease in HR. A shorter distance of the stenosic lesion from the carotid bifurcation (OR = 0.92, 95% CI: 0.87–0.99, *p* = 0.018) was associated with reduced HR, indicating that lesions situated closer to the carotid bifurcation were more frequently associated with significant HR reduction. Hemodynamic measurements obtained in the angiography suite demonstrated significant associations, with a greater decline in systolic blood pressure (ΔSBP; OR = 1.03, 95% CI: 1.01–1.04, *p* < 0.001) and mean arterial pressure (ΔMAP; OR = 1.04, 95% CI: 1.01–1.06, *p* < 0.001) both associated with an increased likelihood of HR depression. In contrast, the specific type of stent utilized (Exact vs. Cguard) demonstrated no significant association with post-procedural HR depression.

The multivariable model demonstrated a statistically significant fit (Omnibus test; *p* = 0.018) and accounted for the outcome variance with a Nagelkerke’s R^2^ of 0.191 and an overall classification accuracy of 67.7.

Multivariate logistic regression analysis (Table 4) showed that a shorter distance from the stenosis to the carotid bifurcation was independently associated with an increased risk of hemodynamic depression (OR = 0.90, 95% CI: 0.82–0.99, *p* = 0.023). Other variables, including hypertension, emergent presentation, degree of stenosis, calcification score, and stenosis length, were not significantly associated with hemodynamic outcomes.

## 4. Discussion

This study evaluated predictors of HI among patients undergoing CAS who were initially classified based on clinical parameters, particularly the absence of markedly low baseline HR or blood pressure, factors that are associated with an increased risk of vagal responses [17,25]. The cohort included both symptomatic (71.2%) and asymptomatic (28.8%) patients. Despite this selection, HI remained frequent, with 49.6% of the cohort experiencing a significant (>20%) reduction in HR during the procedure. These findings suggest that current risk stratification methods may not fully capture susceptibility to autonomic disturbances during CAS, emphasizing the need for refined predictive strategies.

Carotid artery stenting has become an increasingly common and generally safe procedure, with favourable outcomes reported in both symptomatic and asymptomatic patients [20,26]. Nevertheless, the incidence of intra-procedural bradycardia and hypotension remains considerable [15,27]. Although prophylactic atropine can attenuate these hemodynamic disturbances, its universal use is not without risk, as it may precipitate abrupt increases in HR and blood pressure, potentially leading to complications such as myocardial ischemia or arrhythmias [28]. While extreme and refractory cases of post-CAS bradycardia may require advanced interventions such as temporary transvenous cardiac pacing, all patients in our cohort were successfully managed conservatively or with standard pharmacological support, showing transient hemodynamic alterations that fully resolved within the first 24 h. Furthermore, these transient episodes of heart rate depression did not predict or lead to acute peri-procedural complications, such as stent thrombosis or neurological events.

In our analysis, a greater distance between the carotid stenosis and the bifurcation was independently associated with a reduced risk of hemodynamic instability, with each additional millimetre corresponding to an 8% decrease in odds. This observation aligns with prior studies and underscores the anatomical importance of lesion positioning in the pathophysiology of HI during carotid artery stenting [24,27,29,30]. During stent deployment, the highest resistance is encountered at the most stenotic segment of the vessel, leading to concentrated mechanical stress at the site of maximal luminal narrowing. When this segment lies near the carotid bifurcation, particularly within or adjacent to the carotid bulb, where baroreceptors are most densely concentrated, the applied pressure is more likely to provoke a robust parasympathetic reflex, manifesting as bradycardia. In contrast, more distal lesions are less likely to transmit this mechanical force to the baroreceptor-rich zone, thereby diminishing the autonomic response and reducing the likelihood of hemodynamic depression [31]. We also observed that a higher ΔSBP/ΔMAP was associated with HI, indicating that systolic and mean arterial pressures decline proportionally during episodes of bradycardia. This parallel reduction supports the presence of a coordinated reflex response consistent with baroreceptor activation during carotid artery stenting [9,32].

Several prior studies have examined the relationship between lesion location and hemodynamic responses during CAS. Consistent with our findings, Liu et al. reported that a stenosis-to-bifurcation distance of <10 mm was associated with a 2.11-fold higher likelihood of hemodynamic depression compared with distances >10 mm, and that stenotic involvement of the carotid bulb increased this risk by 1.9-fold [18]. Similarly, Choi et al. demonstrated that carotid bulb involvement was an independent predictor of periprocedural bradycardia and persistent postprocedural hypotension [27]. In contrast, Elewa et al. found that broader categorizations of lesion location, such as laterality or CCA versus ICA involvement, did not predict hemodynamic instability [33].

Furthermore, the potential role of statins in modulating hemodynamic stability post-CAS warrants consideration. Beyond lipid-lowering, statins exert pleiotropic effects, including plaque stabilization and improved endothelial function. Growing evidence shows that these mechanisms may protect against protracted hemodynamic depression by enhancing carotid sinus baroreceptor sensitivity [30]. Although baseline statin utilization was not captured in our retrospective database and could not be evaluated, these established mechanisms underscore the importance of perioperative medication profiles in future prospective risk stratification.

A key distinction of this study compared to previous work is the deliberate stratification of patients based on pre-procedural clinical parameters, with the primary analysis focused on patients not meeting established high-risk criteria. By isolating this subgroup, our findings highlight that even patients without traditional risk factors remain susceptible to hemodynamic instability during carotid artery stenting. This underscores the importance of thorough pre-procedural assessment and suggests that predictive factors for HI should be evaluated irrespective of initial risk classification. Furthermore, the potential benefit of prophylactic atropine administration may warrant consideration even among patients without predefined high-risk characteristics.

Notably, we did not observe a higher incidence of bradycardia in emergent procedures, nor across commonly used clinical risk classifications. Although previous studies have identified factors such as smoking, larger balloon diameter (>4 mm), and older age as predictors of hemodynamic instability, these associations were not observed in our cohort [24,29]. This discrepancy may be explained by the intentional exclusion of patients with marked baseline bradycardia, resulting in a population with relatively preserved hemodynamic stability in whom the influence of such clinical predictors may be less pronounced.

This study has several limitations. First, its retrospective design introduces potential sources of bias and limits the capacity to establish causal relationships. Consequently, the observed associations should be interpreted with caution and not inferred as definitive causation. Additionally, due to the retrospective nature of the study, certain baseline clinical and diagnostic characteristics, such as statin use, body mass index (BMI), ASA physical status classification, specific plaque morphology, and structured pre-procedural echocardiographic data, were not systematically recorded. Likewise, patient-reported psychological factors, including baseline anxiety and distress, were unavailable. Consequently, these variables could not be evaluated as potential predictors of post-procedural hemodynamic depression. Furthermore, hemodynamic depression in this study was defined as a >20% reduction in heart rate from baseline, in alignment with commonly used thresholds in previous studies [15,24]. While this threshold is clinically relevant, it may not encompass the full spectrum of clinically significant hemodynamic disturbances. Lastly, this study was limited to intra-procedural and early post-procedural hemodynamic outcomes. Potential long-term consequences of hemodynamic instability, such as delayed cardiovascular events or neurological impairment, were not assessed, limiting insight into the broader clinical significance of these disturbances.

## 5. Conclusions

Hemodynamic instability remains a prevalent and clinically significant complication during CAS, even among patients without predefined high-risk characteristics based on standard pre-procedural assessments. Our findings indicate that lesion-specific anatomical characteristics, particularly proximity to the carotid bifurcation, play a central role in determining hemodynamic responses during the procedure. These findings highlight important limitations in current risk stratification models and suggest that existing criteria may fail to capture key contributors to autonomic instability. The results underscore the need for more nuanced predictive tools to inform targeted prophylactic strategies and improve procedural safety in CAS.

## Figures and Tables

**Table 1:** Baseline characteristics of the study population, with and without significant post-procedural heart rate depression (<20% vs. >20% heart rate decrease).

Variable	HR Decrease < 20% (*N* = 63)	HR Decrease > 20% (*N* = 62)	*p*-Value
Age (years), Mean ± SD	67.3 ± 8.2	67.1 ± 7.8	0.858
Female, *n* (%)	15 (23.8)	18 (29.0)	0.508
Premorbid mRS, median (IQR)	0 (0; 1.25)	0 (0; 1.0)	0.023
Diabetes mellitus, *n* (%)	37 (58.7)	28 (45.2)	0.130
Hypertension, *n* (%)	52 (82.5)	39 (62.9)	0.015
Dyslipidemia, *n* (%)	46 (74.2)	40 (64.5)	0.244
EF > 40%, *n* (%)	60 (95.2)	68 (91.9)	0.456
CIHD, *n* (%)	26 (41.3)	17 (27.4)	0.105
Atrial fibrillation	3 (4.8)	2 (3.3)	0.675
Prior MI, *n* (%)	11 (17.5)	9 (14.5)	0.654
Smoking, *n* (%)	25 (39.7)	29 (46.8)	0.424
COPD, *n* (%)	4 (6.3)	7 (11.3)	0.336
Anemia, *n* (%)	3 (4.8)	3 (4.8)	1.0
PVD, *n* (%)	6 (9.5)	4 (6.5)	0.527
Neck radiation, *n* (%)	2 (3.2)	2 (3.2)	1.0
Prior CEA, *n* (%)	4 (6.3)	1 (1.6)	0.365
Chronic kidney disease, *n* (%)	5 (7.9)	5 (8.1)	0.979
Baseline Troponin, median (IQR)	20.0 (17.0; 25.0)	23.0 (16.4; 33.0)	0.978
Chronic Beta-Blocker therapy, *n* (%)	23 (36.5)	16 (25.8)	0.198
Chronic Ace-Inhibitor therapy, *n* (%)	33 (52.4)	20 (32.3)	0.024
Chronic Diuretic therapy, *n* (%)	2 (3.2)	6 (9.7)	0.157
Chronic CCB therapy, *n* (%)	12 (19.0)	11 (17.7)	0.851

SD, standard deviation; mRS, modified Rankin Scale; IQR, interquartile range; CIHD, chronic ischemic heart disease; MI, myocardial infarction; COPD, chronic obstructive pulmonary disease; PVD, peripheral vascular disease; CEA, carotid endarterectomy; CCB, calcium channel blocker.

**Table 2:** Distributions of anatomic, radiologic and procedural data, with and without significant post-procedural heart rate depression (<20% vs. >20% heart rate decrease).

Variable	HR Decrease < 20% (*N* = 63)	HR Decrease > 20% (*N* = 62)	*p*-Value
Procedure urgency (ref: emergent), *n* (%)	8 (12.7)	13 (21.0)	
Semi-emergent	36 (57.1)	32 (51.6)	0.238
Elective	19 (30.2)	17 (27.4)	0.286
Hypotension (<40% in SBP), *n* (%)	12 (19.0)	38 (61.3)	0.001
IV TPA pre-procedure, *n* (%)	5 (8.1)	6 (9.7)	0.752
If emergent/semi-emergent; NIHSS pre-procedure, median (IQR)	12.0 (7.0; 15.0)	6.0 (3.75; 13.5)	0.532
If emergent/semi-emergent; ASPECTS pre-procedure, median (IQR)	10.0 (10.0; 10.0)	10.0 (8.0; 10.0)	0.156
Woodcock calcification score, median (IQR)	3.0 (2.0; 4.0)	3.0 (2.0; 4.0)	0.885
Left side stent, *n* (%)	34 (54.8)	35 (56.5)	0.857
Cervical level of carotid bifurcation, median (IQR)	4.0 (4.0; 4.0)	4.0 (3.0; 4.0)	0.909
Distance from stenosis to bifurcation (mm), Mean ± SD	5.5 ± 7.3	2.67 ± 4.7	0.018
% of stenosis (NACET criteria), median (IQR)	90.0 (90.0; 95.0)	90.0 (90.0; 95.0)	0.607
Stenosis length (mm), median (IQR)	12.0 (8.0; 17.0)	10.0 (8.0; 13.5)	0.114
Degree of contralateral stenosis per DSA, median (IQR)	10.0 (0; 50.0)	0 (0; 50.0)	0.713
Local anesthesia, *n* (%)	56 (93.3)	51 (82.3)	0.072
Exact stent (vs. Cguard), *n* (%)	57 (90.5)	54 (88.5)	0.723

HR, heart rate; SBP, systolic blood pressure; IV TPA, intravenous tissue plasminogen activator; NIHSS, National Institutes of Health Stroke Scale; IQR, interquartile range; ASPECTS, Alberta Stroke Program Early CT Score; DSA, digital subtraction angiography; mm, millimeter; %, percentage; NACET, North American Carotid Endarterectomy Trial.

**Table 3:** Demographic, clinical and radiological predictors of hemodynamic depression following carotid angioplasty and stenting.

Variable	HR Decrease < 20% (*N* = 63)	HR Decrease > 20% (*N* = 62)	OR	95% CI	*p*-Value
Age (years), Mean ± SD	67.3 ± 8.2	67.1 ± 7.8	1.0	0.95–1.04	0.858
Female, *n* (%)	15 (23.8)	18 (29.0)	1.31	0.59–2.91	0.508
Diabetes mellitus, *n* (%)	37 (58.7)	28 (45.2)	0.58	0.29–1.18	0.130
Hypertension, *n* (%)	52 (82.5)	39 (62.9)	0.36	0.16–0.82	0.015
Dyslipidemia, *n* (%)	46 (74.2)	40 (64.5)	0.63	0.29–1.37	0.244
EF > 40%, *n* (%)	60 (95.2)	68 (91.9)	0.57	0.13–2.50	0.456
CIHD, *n* (%)	26 (41.3)	17 (27.4)	0.54	0.25–1.14	0.105
Prior MI, *n* (%)	11 (17.5)	9 (14.5)	0.80	0.31–2.10	0.654
Smoking, *n* (%)	25 (39.7)	29 (46.8)	1.34	0.66–2.72	0.424
COPD, *n* (%)	4 (6.3)	7 (11.3)	1.88	0.52–6.77	0.336
Chronic kidney disease, *n* (%)	5 (7.9)	5 (8.1)	1.02	0.28–3.71	0.979
Chronic Beta-Blocker therapy, *n* (%)	23 (36.5)	16 (25.8)	0.61	0.28–1.30	0.198
Chronic Ace-Inhibitor therapy, *n* (%)	33 (52.4)	20 (32.3)	0.43	0.21–0.90	0.024
Chronic Diuretic therapy, *n* (%)	2 (3.2)	6 (9.7)	3.27	0.63–16.86	0.157
Chronic CCB therapy, *n* (%)	12 (19.0)	11 (17.7)	0.92	0.37–2.27	0.851
Left-side stent, *n* (%)	34 (54.8)	35 (56.5)	1.07	0.53–2.17	0.857
Exact stent (vs. Cguard), *n* (%)	57 (90.5)	54 (88.5)	0.81	0.26–2.57	0.723
Local anesthesia, *n* (%)	56 (93.3)	51 (82.3)	0.33	0.10–1.11	0.072
Procedure urgency (ref: emergent), *n* (%)	8 (12.7)	13 (21.0)	1		
Semi-emergent	36 (57.1)	32 (51.6)	0.55	0.20–1.49	0.238
Elective	19 (30.2)	17 (27.4)	0.55	0.18–1.65	0.286
Pre-procedure ASPECTS (emergent cases only), median (IQR)	10.0 (10.0; 10.0)	10.0 (8.0; 10.0)	0.51	0.20–1.33	0.168
Cervical level of carotid bifurcation, median (IQR)	4.0 (4.0; 4.0)	4.0 (3.0; 4.0)	0.97	0.56–1.68	0.909
Distance from stenosis to bifurcation (mm), Mean ± SD	5.5 ± 7.3	2.67 ± 4.7	0.92	0.87–0.99	0.018
% of stenosis (NACET criteria), median (IQR)	90.0 (90.0; 95.0)	90.0 (90.0; 95.0)	1.01	0.97–1.06	0.607
Woodcock calcification score, median (IQR)	3.0 (2.0; 4.0)	3.0 (2.0; 4.0)	0.98	0.79–1.23	0.885
Stenosis length (mm), median (IQR)	12.0 (8.0; 17.0)	10.0 (8.0; 13.5)	0.94	0.88–1.01	0.114
Degree of contralateral stenosis per DSA, median (IQR)	10.0 (0; 50.0)	0 (0; 50.0)	1.0	0.99–1.01	0.713
ΔSBP measurement, mean ± SD	51.7 ± 30.4	74.7 ± 27.6	1.03	1.01–1.04	<0.001
ΔMAP measurement, mean ± SD	35.0 ± 19.4	47.6 ± 18.1	1.04	1.01–1.06	<0.001

HR, heart rate; OR, odds ratio; CI, confidence interval; SD, standard deviation; EF, ejection fraction; CIHD, chronic ischemic heart disease; MI, myocardial infarction; COPD, chronic obstructive pulmonary disease; CCB, calcium-channel blocker; ASPECTS, Alberta Stroke Program Early CT Score; IQR, interquartile range; DSA, digital subtraction angiography; NACET, North American Carotid Endarterectomy Trial; SBP, systolic blood pressure; MAP, mean arterial pressure.

**Table 4:** Binary logistic regression model assessing the risk of hemodynamic depression in patients undergoing carotid angioplasty and stenting.

Variable	OR	95% CI	*p*-Value
Hypertension	0.45	0.17–1.18	0.105
Emergent (vs. Elective)	0.80	0.30–2.10	0.643
% Stenosis	1.01	0.95–1.08	0.775
Calcification score	1.27	0.93–1.72	0.128
Distance from stenosis to bifurcation	0.90	0.82–0.99	0.023
Stenosis length	0.93	0.86–1.01	0.098

## Data Availability

The datasets generated and/or analyzed during the current study are not publicly available due to patient confidentiality and institutional policies.
